# Retraction: Radio frequency triggered curcumin delivery from thermo and pH responsive nanoparticles containing gold nanoparticles and its *in vivo* localization studies in an orthotopic breast tumor model

**DOI:** 10.1039/d0ra90082f

**Published:** 2020-08-04

**Authors:** N. Sanoj Rejinold, Reju George Thomas, Muthunarayanan Muthiah, K. P. Chennazhi, In-Kyu Park, Yong Yeon Jeong, K. Manzoor, R. Jayakumar

**Affiliations:** Amrita Centre for Nanosciences and Molecular Medicine, Amrita Institute of Medical Sciences and Research Centre, Amrita Vishwa Vidyapeetham University Kochi-682041 India rjayakumar@aims.amrita.edu jayakumar77@yahoo.com +91 484 2802020 +91 484 2801234; Department of Radiology, Chonnam National University Hwasun Hospital, Chonnam National University Medical School Gwangju 501-746 South Korea; Department of Biomedical Science, BK21 PLUS Center for Creative Biomedical Scientists, Chonnam National University Medical School Gwangju 501-746 South Korea pik96@chonnam.ac.kr

## Abstract

Retraction of ‘Radio frequency triggered curcumin delivery from thermo and pH responsive nanoparticles containing gold nanoparticles and its *in vivo* localization studies in an orthotopic breast tumor model’ by N. Sanoj Rejinold *et al.*, *RSC Adv.*, 2014, **4**, 39408–39427, DOI: 10.1039/C4RA05727A.

We, the named authors, hereby wholly retract this *RSC Advances* article due to duplication of images in Fig. 9–12, 15, 18 and 19, which affect the reliability of the data and the overall conclusions presented in the published article.

Signed: N. Sanoj Rejinold, Reju George Thomas, Muthunarayanan Muthiah, K. P. Chennazhi, In-Kyu Park, K. Manzoor and R. Jayakumar, 24th July 2020.

Yong Yeon Jeong was contacted but did not respond.

Retraction endorsed by Laura Fisher, Executive Editor, *RSC Advances*.

## Supplementary Material

